# Chitin and Chitosan: Prospective Biomedical Applications in Drug Delivery, Cancer Treatment, and Wound Healing

**DOI:** 10.3390/md20070460

**Published:** 2022-07-17

**Authors:** Parnian Baharlouei, Azizur Rahman

**Affiliations:** 1 Centre for Climate Change Research, University of Toronto, ONRamp at UTE, Toronto, ON M5G 1L5, Canada; parnian.baharlouei@mail.utoronto.ca; 2 Physiology and Human Biology, University of Toronto, Toronto, ON M5S 1A8, Canada; 3 A.R. Environmental Solutions Inc., ICUBE-University of Toronto, Mississauga, ON L5L 1C6, Canada

**Keywords:** chitin, chitosan, wound healing, drug delivery, cancer drugs, tissue engineering, nanobiotechnology

## Abstract

Chitin and its derivative chitosan are highly abundant polymers in nature, appearing in both the shells and exoskeletons of various marine and non-marine species. Since they possess favorable properties, such as biocompatibility, biodegradability, non-toxicity, and non-immunogenicity, they have gained recent attention due to their enormous potential biomedical applications. The polycationic surface of chitosan enables it to form hydrogenic and ionic bonds with drug molecules, which is one of its most useful properties. Because chitosan is biocompatible, it can therefore be used in drug delivery systems. The development of chitosan-based nanoparticles has also contributed to the significance of chitin as a drug delivery system that can deliver drugs topically. Furthermore, chitin can be used in cancer treatment as a vehicle for delivering cancer drugs to a specific site and has an antiproliferative effect by reducing the viability of cells. Finally, chitosan can be used as a wound dressing in order to promote the faster regeneration of skin epithelial cells and collagen production by fibroblasts. As discussed in this review, chitin and chitosan have diverse applications in the medical field. Recognizing the biomedical applications of these two polymers is essential for future research in tissue engineering and nanobiotechnology.

## 1. Introduction

**Figure 1:**
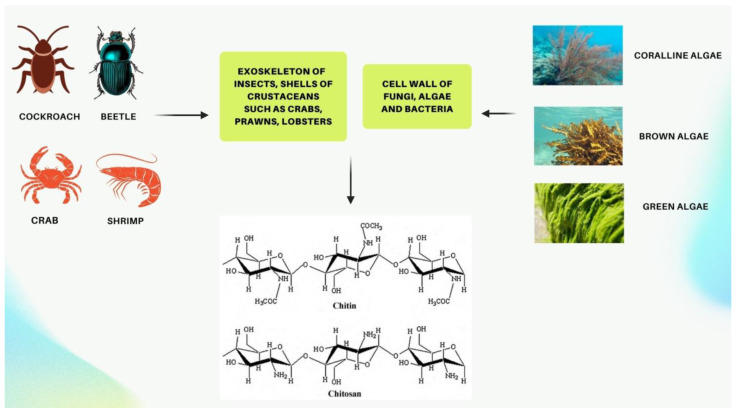
Sources of chitin and the structure of chitin and chitosan. [Created with BioRender.com accessed on 10 May 2022].

Chitin is a polysaccharide composed of crystallized N-Acetyl D-glucosamine monomers and 1–4 glycosidic bonds [1,2]. This structural polymer is highly abundant in the shells of marine crustaceans, cell walls of various organisms such as fungi, coralline algae, green and brown algae, and bacteria, as well as the exoskeleton of crustaceans, molluscs, and insects (Figure 1) [3,4,5,6,7,8,9]. In 2014, the first evidence of chitin inside the cell walls of the coralline algae *Clathromorphum compactum* was observed, and chitin was found to play an important role in the calcification process of this marine species [4]. Initially named fungine in 1811, it was subsequently renamed chitin in 1823 [10]. It occurs in about 19 animal phyla, bacteria, algae, and fungi, with a production rate of 10^11^–10^14^ tons per year [10]. Although chitin is present in nature, multiple tons of it transform into marine shell wastes or other types of industrial wastes [11]. Chitin has recently gained much attention from scientists due to its important properties, which include nontoxicity, biocompatibility, and biodegradability, the ability to degrade by enzymes, enabling it to be used in many biomedical applications, including drug delivery, tissues engineering, wound healing, and cancer therapy. [11]. In spite of being the second most abundant polymer after cellulose, chitin is still largely unutilized because its semi-crystalline structure with a large number of intermolecular hydrogen bonds causes it to be insoluble in most solvents [6,12]. With the advent of new technologies, however, the partial N-deacetylation of chitin can now be converted into a soluble substance called chitosan. The deacetylation of extracted chitin is largely carried out in strong alkaline media, resulting in chitosan becoming soluble in acidic environments [13]. Hence, chitosan is a synthetically deacetylated form of chitin that can be used in the food industry as an edible and biodegradable film for packaging food, food preservations, pharmaceuticals, water and wastewater treatment, cosmetics, wound dressings, and many other applications [1,6,11,14,15]. 

**Figure 2:**
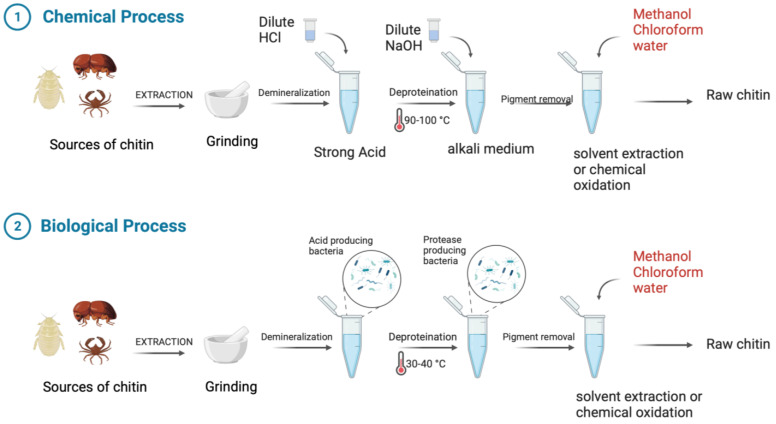
Chemical and biological processes to isolate chitin from animal sources. [Created with BioRender.com accessed on 10 May 2022].

As mentioned earlier, the shells of crustaceans and shrimps, insect exoskeletons, and the cell walls of fungi such as aspergillus and mucor are the main sources of chitin and chitosan [16]. A large scale of industrial chitin production is derived from crab shells and shrimp shells, so recycling these wastes will yield valuable byproducts that can be used in medical and pharmaceutical applications [16]. To prepare chitin, the first step is to extract it from shellfish wastes. Shellfish wastes contain chitin, lipids, inorganic salts, and proteins as major components. Hence, chitin is extracted from these structural components using two common methods: chemical and biological processes (Figure 2) [17,18,19]. The purpose of these processes is to isolate chitin from minerals such as calcium carbonate and calcium phosphate and proteins and lipids or other macromolecules embedded in shells and cell walls [17]. In strong alkaline (>1 M NaOH) and acetic (>3 M HCl) environments combined with heat, chitin can be transformed into its deacetylated form, chitosan, or even partially fragmented [20]. Therefore, sulfuric acid, nitric acid, formic acid, acetic acid, and in particular, dilute hydrochloric acid are commonly used in the demineralization process of chitin [17,18,21]. The separation of proteins and pigments such as carotenoids is performed using dilute sodium hydroxide and acetone (or other organic solvents), respectively [17,18]. As an alternative method, biological processes may provide more desirable results due to their relatively low cost and environmental friendliness, low energy consumption, and reproducibility. They can also produce chitin with a higher molecular weight and better crystal structure [17]. Biological processes rely largely on enzymatic reactions and microbial fermentation [17,22]. However, the basis of these reactions, such as with the chemical process, is the production of acidic and alkaline products but from microorganism fermentation and enzymatic metabolites [17]. 

Though chemical processes make chitin extraction easier and more efficient, they have some drawbacks when used on an industrial scale with industrial protocols. There are two main concerns about conventional methods of chitin extraction: the use of strong chemicals such as HCl, and the lower quality of raw extracted chitin due to contamination [15]. Therefore, obtaining high-quality chitin involves sophisticated instrumentation such as the use of ultrafiltration and molecular sieving. However, these techniques are costly, which creates significant financial barriers, especially for developing countries. Recent efforts are underway to develop novel methods for chitin extraction. As an example, Tissera et al. introduced a new process called “pretreatment” for the extraction of chitin from blue swimmer crab shell waste, proposing that this procedure helped improve the quality of extracted chitin for large-scale production [15]. They soaked crushed crab shell pieces in acetic acid (with concentrations of 0.05, 0.10, and 0.50 M) and citric acid with concentrations of 0.05, 0.10, and 0.50 M and found that acetic acid at 0.5 M resulted in the better cleaning of crab shells. They found that this pre-acid treatment helped the rigid structure of shells to become more flexible as well as slightly beginning the process of demineralization [15]. The results showed that this treatment with 0.5 M acetic acid helped remove any remaining muscle tissue that remained, which led to a lower concentration of NaOH being required for the deproteinization of crab shells. Furthermore, this pretreatment led to a lower concentration of acid required for the demineralization step since acetic acid aids in calcium carbonate removal [15].

Traditional chitin extraction methods have been thought of as time-consuming and expensive, with production times greater than one day. Therefore, other methods aimed at speeding up the process of chitin extraction have been studied in recent years. Kaya et al. proposed to treat the shells of crustaceans such as crabs, crayfish, and shrimp with two NaClO treatments (10 min each) before the main processes of demineralization and deproteinization to ensure the complete disintegration and removal of pigments [23]. They found that by introducing NaClO, they were able to extract chitin in a shorter amount of time while the yield of chitin was similar to conventional extraction, and the color of extracted chitin was white, as in commercially available chitin [23]. The elemental analysis, FT-IR, and XRD of extracted chitin suggested that it has a high similarity to commercial chitin; consequently, they suggested that this new treatment of chitin should be considered for extracting chitin from crab shells, crayfish shells, and shrimp shells [23].

In another innovative study, Machałowski et al. isolated chitin from Caribena versicolor spider molt cuticles [24]. Arachnoids have a mineral-free cuticle, so demineralization (such as decalcification) can be skipped, and chitin isolation can begin with deproteinization and depigmentation [24]. Their study included removing pigments as well as chitin from spider cuticles using microwave-assisted methods (MWI) [24]. In the first step, which involved removing lipids and waxes from the cuticle, the cuticles were exposed to microwave radiation while being treated with chloroform and ethanol in a ratio of 2 to 1 [24]. In the second step, they used microwave radiation alongside NaOH treatment to remove other proteins and pigments from the cuticle [24]. The final step was to treat the cuticles with H_2_O_2_ under microwave radiation in order to remove any residual pigments and obtain tubular chitin [24]. The bodies of C. versicolor spiders are covered with a strong exoskeleton (cuticle) consisting of an inner layer with chitin and proteins and an outer layer (without chitin) [24]. Due to this, these spiders lose a large number of chitin-rich cuticles during their molting cycle; therefore, tubular chitin from their cuticles could be used as a scaffold-based catalyst and tissue engineering material [24].

## 2. Chitosan as a Possible Drug Delivery Agent

The term “drug delivery systems” (DDS) refers to products that combine an active pharmaceutical agent with a suitable carrier—typically a polymer—and have been used in the pharmaceutical industry to improve the therapeutic potential and bioavailability of drugs [1,25]. By using hydrogels, scaffolds, micro- and nano-particles, and organic or inorganic matrices as carriers of active drug agents, this system delivers drugs in a sustained and controlled manner [1,10]. Among these, hydrogels, due to their chemical stability, are able to protect and maintain the encapsulated drug in extreme environmental conditions, and by dissolving in body fluids, lead to the release of the drug at the desired site and with the required concentration [1]. Controlled delivery is a basic property of the ideal DDS, which is used to maintain the plasma concentration of the active drug at a constant rate and within the therapeutic window [25]. Another important feature of DDS is targeted drug delivery or directing the active substance to a specific site [25]. In DDS, different types of vehicles such as tablets, capsules, and hydrogels are commonly used, depending on the target site and the route of administration [1]. The delivery of drugs can be performed either through invasive or non-invasive routes, such as parenteral and mucosal administration, respectively [25]. Mucosal drug delivery includes oral, buccal, nasal, pulmonary, and rectal routes, and most DDS use this route of administration to enhance mucoadhesiveness and, consequently, increase drug release [25]. 

A number of studies have been conducted on chitin and chitosan to determine their potential as drug delivery agents. Chitosan, for example, is significantly utilized in the production of hydrogels for drug delivery due to its valuable properties, including bioadhesion, having a polycationic surface that facilitates the creation of hydrogenic and ionic bonds, and biocompatibility, meaning that in contact with living tissue or body fluids, it does not produce any toxins or elicit an immune response [1,2]. 

Marine sponges provide a good source of naturally occurring chitin that can be used for drug delivery by extracting chitin from their skeletons [26]. Kovalchuk et al. examined the potential application of fabric-like chitin scaffolds from the demosponge *Ianthella flabelliformis* for the first time [26]. The researchers found that these chitin scaffolds possess three-dimensional (3D) matrices that retain antiseptic decamethoxine absorbed from a 0.1% ethanol solution [26]. Moreover, they demonstrated that decamethoxine diffused from these mesh-like scaffolds into an agar diffusion assay and inhibited the growth of Staphylococcus aureus, confirming the potential use of pure chitinous scaffolds in drug delivery [26]. In another study, Zheng et al. prepared 10–100 μm carboxymethyl chitin microspheres (CMCH-Ms) and then encapsulated them in thermosensitive hydroxypropyl chitin hydrogels (HPCH) [27]. To maintain the spherical structure of microspheres with porous microstructures, they used physical cross-linking procedures and increased the temperature in an aqueous two-phase system without using cross-linking agents. Using the physical cross-linking method, carboxymethyl chitins are able to form microspheres at high temperatures because of their high acetylation levels, which make them temperature sensitive [27]. Similarly, hydroxypropyl chitin (HPCH) is also temperature sensitive, and it is capable of reversibly converting into solid hydrogels at elevated temperatures, enabling the in situ formation of hydrogels [27]. Two drugs, naproxen (NPX) and ropivacaine (Ropivacaine), were analyzed in vitro for their loading and release capacities into CMCH-Ms and HPCH gel scaffolds [27]. They found that CMCH-Ms/HPCH gel scaffolds sustained in vitro drug release over a longer period and caused fewer bursts of drug release. Therefore, they determined that drug-loaded CMCH-Ms/HPCH gel scaffolds could be used as a means of delivering local drugs [27]. 

Another technology that has attracted attention in recent years in the drug delivery industry is the use of nanomaterials, which include objects with a size of 1–100 nm [2,28]. Due to the size of these particles, they have a special feature as they follow the rules of quantum mechanics [28]. Nanotechnology can be used for accurate and precise imaging and drug delivery [2,29]. One method of drug delivery is the use of chitosan-based nanoparticles, which are used greatly in the mucosal route to transport the drug to the brain and eyes, as well as to treat diseases such as cancer and gastrointestinal and pulmonary diseases [29,30]. The solubility, diffusion, and size of chitosan-based nanoparticles determine the rate of drug release [31]. Most chitosan-based nanoparticles have a size between 100 and 400 nanometers. Photon correlation spectroscopy can be used to determine the size of the nanoparticles by measuring their Brownian motion [31]. Chitosan nanoparticles are stabilized based on several physical and chemical factors, including agglomeration, coagulation, temperature, the molecular weight of the polymer, and the pH of the medium [31].

In many eye diseases, drugs are used superficially on the ocular surface. However, due to defense mechanisms, less than 5% of drugs used topically are able to cross the cornea and reach intraocular tissue [32,33]. As a result, chitosan-based nanoparticles could be considered a possible solution to increase bioavailability and prolong drug bioavailability in the ocular tissue [32,33,34]. In a study by Silva et al., using nanotechnology, eye drops containing polymer-based nanoparticles consisting of the antibiotic ceftazidime were used to treat ocular infection with *pseudomonas aeruginosa*, and the degree of mucoadhesion or the interaction between nanoparticles delivered through the eye and ocular mucosal tissue was later analyzed [32]. The results of their study demonstrated that the use of polymeric-based nanoparticles facilitated the transport of antibiotics through the mucosal barrier of the eye as it enhanced the bioavailability of the drug and protected against degradation [32]. In addition, they were able to show that chitosan, due to its structural properties, increases the contact time between antibiotics and ocular mucosal tissue, so that a strong electrostatic interaction between the amine group of chitosan and salicylic acid, which is a main component of mucin, causes the drug to stay inside the eye longer. Other properties of chitosan have demonstrated its potential for drug delivery systems, including hydrophilicity, biodegradability, and antimicrobial activity [32].

In an innovative study, Cánepa et al. investigated the efficacy of interferon-alpha (IFNα) delivery using chitosan-based nanoparticles through oral administration [35]. It has been known that the use of drugs via the oral route could reduce the absorption of drugs into the gastrointestinal tract due to the degradation by brush border enzymes and hepatic metabolism [35,36,37]. As a result, many pharmaceutical companies are trying to increase the bioavailability of prescribed orally-administered drugs [37]. IFNα is used as a drug to treat cancer and viral infections; however, due to its short half-life, it usually needs to be taken regularly by patients, and this regime could have its own side effects and drawbacks [38,39,40,41,42]. Cánepa et al. showed that the antiviral effect of encapsulated IFNα by chitosan-based nanoparticles was comparable to commercial IFNα on the market [35]. Furthermore, the bioavailability of the drug improved within the gastrointestinal tract due to the electrostatic interaction between the chemical groups in chitosan and salicylic acid in mucin, which led to the increased size of nanoparticles following the adsorption of mucin, as well as decreased surface charge of nanoparticles [35].

## 3. Application of Chitin and Chitosan in Cancer Therapy

Cancer causes 13% of deaths worldwide, and the rate of cancer sufferers is increasing [43]. According to estimates, 12 million people will have cancer by 2030, with breast cancer, colorectal cancer, lung cancer, and prostate cancer being the most common types [43]. As part of anti-cancer therapy, it is crucial to use carriers for drugs that are safe and can deliver the drugs efficiently to the cancerous tissue without causing harm to healthy cells [43]. There are many types of drug delivery vehicles, but polymeric vehicles have gained much attention because not only do they increase drug targeting ability, but they also improve blood retention time by preventing drugs from being excreted in the urine [44]. Chitosan and chitin have been used as drug delivery carriers in chemotherapy. 

In one example by Dev et al., carboxymethyl chitin (CMC) nanoparticles, soluble in water and prepared by cross-linking with CaCl_2_ and FeCl_3_, were loaded with the anti-cancer drug, 5-fluorouracil (5-Fu), using an emulsion cross-linking procedure [45,46]. The chemical modification of chitin and chitosan has been shown to greatly affect their solubility in water, physiological properties, biodegradability, as well as their reactivity with other molecules [47]. It is, therefore, essential to find ways that can maximize chitin and chitosan’s solubility in common solvents in order to make use of them. Chitin carboxymetylation has been previously shown to enhance the water solubility of chitin while showing biocompatibility and no antibody induction [47,48]. Using monochloroacetic acid and isopropyl alcohol as a solvent, they are prepared by a condensation reaction between chitin powder and monochloroacetic acid [47]. Dev et al. determined that the nanoparticles were not toxic to normal fibroblast cells in a mouse model and that the drug was released in a controlled and sustained manner [46]. In addition, CMC nanoparticles exhibited antibacterial activity and ferromagnetic properties that can be used to deliver drugs using tracking systems [46]. Due to their favorable properties, carboxymethyl chitin can also be used as a wound dressing material; however, it must be cross-linked in order to maintain its integrity [47].

Chitosan polymers have been used in different forms, such as in hydrogels, nanoparticles, and nanofibers, to deliver drugs in the treatment of a variety of cancer types. Chitosan polymers boast versatile properties, making them great candidates for targeting chemotherapy in the treatment of numerous cancers. Different features can be achieved by changing the surface moieties, such as including a membrane-penetrating peptide, monoclonal antibodies, or surface receptors targeting specific cancer cells [44]. Below are a few examples of recent attempts to use this polymer to deliver drugs in the treatment of a variety of cancers. 

**Figure 3:**
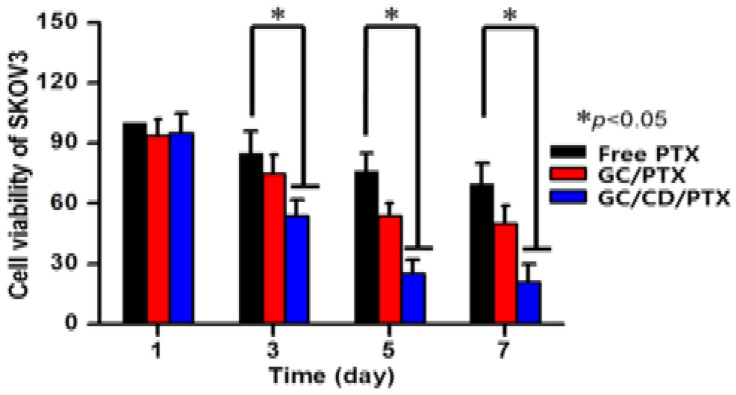
Changes in the cell viability of in vitro SKOV3 cell lines at 1-, 3-, 5- and 7-days post-treatment with PTX only, glycol chitosan hydrogel (GC) combined with PTX (GC/PTX) and GC/PTX complexed with beta-cyclodextrin (β-CD) (GC/CD/PTX). The error bars show mean ± SD with *n* = 3 and * *p* <0.05 [52].

In ovarian cancer, finding a suitable drug delivery system is significantly important because ovarian cancer is the leading cause of death in women, with a 30% chance of 5-year survival in the advanced stages of the disease [49]. Traditional paclitaxel (PTX) chemotherapy, which is the primary drug of choice for chemotherapy, does not have the ability to target cancer with high specificity and, in addition, causes high toxicity in normal cells [49,50,51]. Therefore, using a drug delivery system with high efficiency and specificity could greatly improve the treatment process of the disease. In one study by Hyun et al., an injectable photo-cured glycol chitosan (GC) hydrogel was used to deliver a paclitaxel (PTX)- complexed beta-cyclodextrin (β-CD) drug to assess ovarian cancer in a mouse model of cancer as well as human ovarian tumor cell lines [52]. In addition to demonstrating that the use of GC/CD/PTX and GC/PTX results in sustained drug release in vivo, one of the important results they showed was an increase in the water solubility of PTX by combining it with chitosan-based hydrogel, which, after seven days of post-treatment, decreased the viability of SKOV3, one of the human ovarian tumor cell lines, by 3.33 fold, compared to a cell culture treated only with free PTX (Figure 3) [52,53].

**Figure 4:**
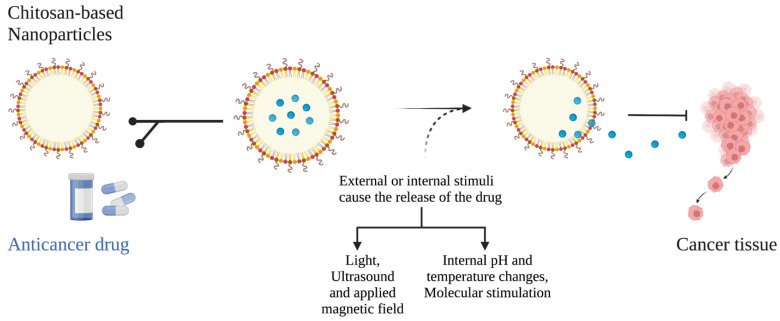
Applications of chitosan-based nanoparticles in tumor-targeted drug delivery. [Created with BioRender.com accessed on 10 May 2022].

Among the many types of carriers, chitosan-based nanoparticles have favorable properties, such as being cost-effective, biodegradable, biocompatible, eco-friendly, and easy to absorb during oral administration. This makes them suitable as carriers of drugs in cancer treatment [43,54,55,56]. Nanocarriers have tunable properties, including modifiable surface functionalities and by altering the surface of these small particles, such as adding a metal, their targeting specificity can also be greatly enhanced [44]. A nanoparticle can contain metal or can be made without it. Although metallic and non-metallic nanoparticles may be effective in targeting cancerous cells, non-metallic particles are preferred for their relative safety and biodegradability. Nanoparticles based on chitosan can be used to deliver drugs via oral, nasal, ocular, and intravenous routes [43]. Due to the spherical structure of nanoparticles, hydrophobic drugs can be assembled inside the inner core of chitosan-based nanoparticles, while hydrophilic drugs can be attached directly to the surface (Figure 4). In recent years, many studies have explored the potential for using drug-loaded chitosan nanoparticles to treat cancers of various types. A study by Asiri et al. examined the potential impact of chitosan-based nanoparticles conjugated with glutaraldehyde (ChNP-GA) on the survivance of human colorectal carcinoma cells (HCT-116) [57]. In this study, the researchers discovered that ChNP-GA significantly reduced the viability of HCT-116 cancer cells in a dose-dependent manner, as well as the cell morphology of the cancer cells (including nuclear condensation and nuclear augmentation) [57].

In an effort to find suitable carriers for cancer therapy, Vijayakumar et al. extracted chitin from shrimp shells to stabilize silver nanoparticles (AgNP) and to treat human hepatocellular carcinoma [58]. Ag-nanoparticles have been used for cancer treatment due to their toxicity to human cells. Their drawback is that they can damage normal cells in the body at the same time. Therefore, the use of AgNP to treat cancer requires the use of a carrier that can access cancer cells only [58]. Chitin, as an appropriate carrier, can be used to deliver AgNP in addition to its anti-cancer properties by inhibiting anti-apoptotic genes [58]. Chitin and chitosan, as safe biological sources, can both be effective in treating cancer by drug delivery and have been shown to have anti-proliferative effects in some human cancers, including colon and lung cancers, which are two of the most common cancers in the world [59,60,61]. Colon cancer is the third most common cancer in the American population, and chemotherapy, known as a promising therapy, faces problems such as increased resistance to chemotherapy drugs [62]. In a study by Santhana Panneer et al., chitin was extracted from *Artemis* cysts, chitin and chitosan-loaded gold nanoparticles were prepared, and their anti-cancer properties were evaluated in two common colon and lung cancers, HT-29 and A549 cell lines, respectively [59]. Their experiments showed that both chitin- and chitosan-based Au nanoparticles could reduce the viability of colon and lung cancer cells, and the percent viability reduction also depended on the concentration of nanoparticles [58]. Essentially, Au+ ions inhibit cell viability by triggering the oxidative stress response in cells, which in turn leads to DNA damage and apoptosis [59].

**Figure 5:**
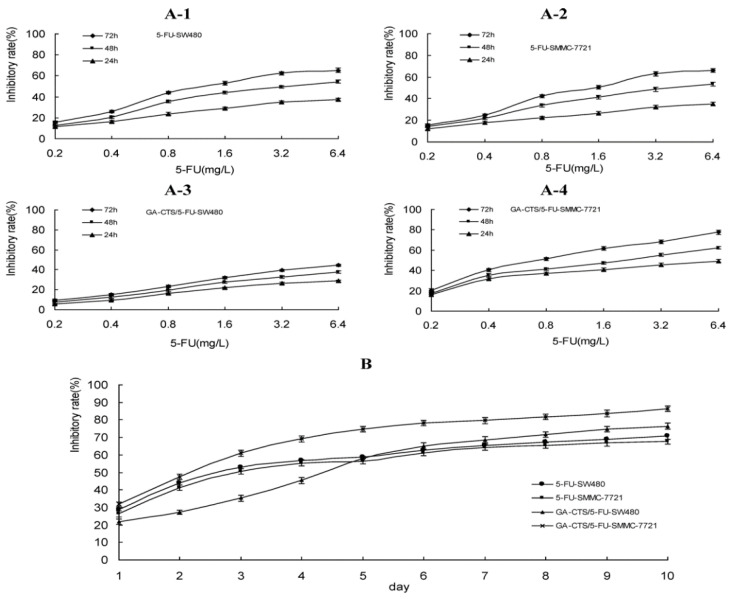
GA-CTS/5-FU and 5-FU cytotoxicity on mouse liver cells SMMC-7721 and SW480 measured by methyl thiazolyl tetrazolium (MTT) assays. (**A1**–**A4**) GA-CTS/5-FU and 5-FU inhibition after 24, 48, and 72 h in vitro are observed by varying the concentration of 5-FU (mg/L). (**B**) The same treatment with 1.6 mg/L of 5-FU after 1 to 10 days. Data are presented as mean ± SD (*n* = 3) [63].

An approach to controlling the release of the anti-cancer drug 5-fluorouracil (5-FU), which inhibits cell proliferation, was demonstrated with the use of chitosan (CTS) as a drug delivery vector in a mouse model of liver cancer [63]. Since the receptor for Glycyrrhetinic acid (GA) is mostly found on the liver cell membrane, Cheng et al. used GA to target liver cells during drug release [63]. To test the dose and time dependency of the cytotoxic effect of 5-FU on liver cells, they prepared a glycyrrhetinic acid-modified chitosan (GA-CTS)-based 5-FU nanoparticle and tested its inhibitory capacity on SMMC-7721 and SW480 cells (Figure 5) [63]. After measuring the inhibitory effect of GA-CTS/5-FU and 5-FU on SMMC-7721 and SW480 cells using different concentrations of 5-FU, they found that the maximum inhibition rate was associated with GA-CTS/5-FU after 24, 48, and 72 h (Figure 5A1–A4). Moreover, while GA-CTS/5-FU showed lower cytotoxicity compared to 5-FU between days 1 and 5, the study found that GA-CTS/5-FU showed a longer duration of activity as it led to a higher inhibitory rate between days 6 and 10 (Figure 5B) [63].

## 4. Potential for Chitin and Chitosan in Wound Healing

The skin tissue that covers the entire surface area of the body protects it from physical, mechanical, and chemical damages, as well as pathogenic microorganisms [64]. Several factors can damage the skin barrier directly or indirectly, including trauma, ulcers, and diseases such as diabetes, resulting in the loss of water, proteins, and an increased risk of bacterial infections [65]. Damage to the dermis of the skin, especially when caused by bacterial infections, may prolong the healing process because dermal cells do not migrate as quickly, and collagen synthesis is delayed [65]. Wound healing is a process that involves the formation of granulation tissue and scar tissue [64]. A key part of wound healing occurs when stem cells are activated, which secrete cytokines and growth factors after they are recruited to the wound [64]. Despite the fact that skin can heal itself if the damage is not severe, there are instances when wound healing takes time or does not bring the appropriate structural integrity [66]. Therefore, various biomaterials can be used to engineer skin tissue through wound dressings. A functional wound dressing has a porous structure that allows oxygen to pass through as well as forming a good barrier against the outside environment [67]. A wound dressing can also speed up wound healing by reducing bleeding and keeping the wound moist [67]. 

The research on wound healing procedures has largely centered on the use of hydrophilic polymeric hydrogels, which act similarly to the extracellular matrix by retaining large amounts of water [66]. In addition to being a renewable source of biomass, chitin and its derivatives have numerous positive properties, including being biocompatible, biodegradable, and able to act as a bioactive material for wound healing [66]. They have also been shown to improve hemostasis by stimulating collagen production in fibroblasts, which contributes to faster wound healing [68]. Chitosan’s hemostatic effects are also attributed to the formation of coagulum with red blood cells and its effect on blood coagulation [67]. Polymers derived from chitin can be combined with other polymers to make a wound dressing that promotes cell proliferation and protects against infection [69]. Additionally, chitin-derived polymers have anti-infection properties, boost growth factors, and promote regeneration and migration of cells, making these polymers suitable for wound dressings [69].

A study by Huang et al. produced nonwoven chitin fabrics using wet chitin fibers for the first time [70]. In the first step, chitin was dissolved in a NaOH–urea solution and then processed by freezing–thawing to produce wet chitin fibers [70]. In their experiments, chitin solution concentrations ranging from 4% wt. to 7% wt. resulted in fibers with high mechanical strength [70]. This chitin nonwoven fabric, applied as a wound dressing, showed a higher level of air permeability and water absorption than regular gauze. Then chitin nonwoven fabrics and gauze were applied to a rabbit’s full-thickness wound model [70]. The results showed that, compared with gauze, chitin nonwoven fabric increased the wound healing speed and caused the wound size to be smaller. Moreover, the average post-wounding day for traditional gauze (16 days) was higher than for chitin nonwomen fabric (12.5 days) [70]. In addition, after 12 days of post-wound treatment with chitin fabric, the wound completely recovered, while that was not the case with gauze [70]. Different mechanisms have been proposed for how chitin-based wound dressing can accelerate wound healing. However, the most well-known mechanism might be related to the ability of chitin and chitosan to reduce inflammatory activity. Chitosan has been shown to activate macrophages, and this activation results in the increased production of growth factors, cytokines, and macrophage inflammatory protein-2 (MIP-2) [71]. These mechanisms lead to higher collagen production, an antibacterial effect, and the proliferation of epithelial tissue is stimulated by MIP-2 [71].

**Figure 6:**
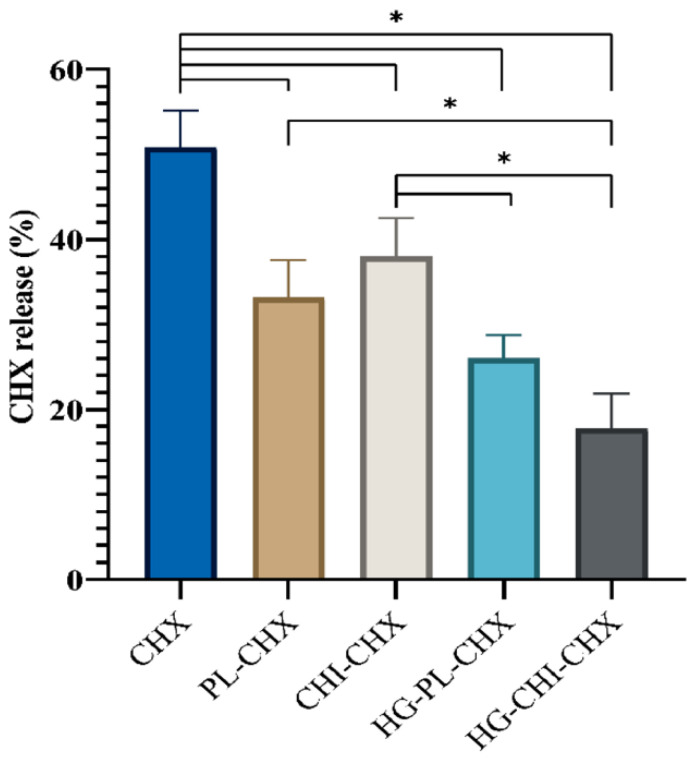
The percentage release (based on the initial concentration) of formulated and free CHX at 32 °C after 24 h when using the Franz diffusion system. CHX = dissolved CHX, PL-CHX = plain, CHX-vesicles, CHI-CHX = CHX-chitosomes, HG-PL-CHX = plain, CHX-vesicles in hydrogel, HG-CHI-CHX = CHX-chitosomes in hydrogel. Since dissolved CHX permeated faster, it showed the greatest percent release. Data are presented as means ± SD (*n* = 3). * *p* < 0.05 [73].

Chitosan has proven antimicrobial properties, which enable it to be used in combination with antimicrobial drugs in the treatment of acute skin injuries [72,73]. Topical skin diseases, however, require lipid-based vehicles such as liposomes because they have a better interaction with the skin and they can deliver drugs that are less soluble in the skin [73]. Liposomes, therefore, can be coated with chitosan to enhance antimicrobial activity during the treatment of skin and soft tissue infections (SSTIs) [73]. In a study by Hemmingsen and colleagues [73], liposomes containing antimicrobial drugs, Chlorhexidine (CHX), were incorporated into chitosan-based hydrogels to enhance liposome retention during drug release. It was found that chitosomes (chitosan-infused vesicles) incorporated with CHX cause a greater release of CHX and sustained therelease of CHX than the CHX formulations without chitosomes (Figure 6) [73]. As such, this will provide insight into the role of chitosan in the long-term retention of drugs during the treatment of skin injuries.

## 5. Concluding Remarks

Chitin is the most abundant polymer after cellulose, and it can be derived from the shells of crustaceans, fungi cell walls, coralline algae, and bacteria. Since chitin and its deacetylated form, chitosan, possess suitable inherent biological and chemical properties, including biocompatibility, biodegradability, bioadhesion ability, nontoxicity, and nonimmunogenicity, they can be used in a wide range of biomedical applications such as drug delivery through mucosal routes. It has been shown that drug-loaded chitin and chitosan can release drugs through long-term sustained release, which contributes to better achieving the continuous drug release needed to maintain therapeutic windows. In addition, nanotechnology has allowed scientists to deliver drugs topically using chitin and chitosan nanoparticles. Hence, nanoparticles can be loaded with drugs to increase their efficiency in reaching the target site. Cancer targeting and wound healing have also been made possible by these molecules due to their high potential. A combination of chitosan hydrogel and chitin nanoparticles may deliver anti-cancer drugs to cancer cells and inhibit their proliferation. In light of the promising applications for nanofibers in tissue engineering, it would be beneficial to focus on developing chitin derivative fibers to serve as biological substitutes for body tissues at the nanoscale.
